# Staged Eye-Plaque Brachytherapy: A Novel Approach for Large Uveal Melanoma

**DOI:** 10.1016/j.adro.2021.100712

**Published:** 2021-05-17

**Authors:** Neil Chevli, Amy C. Schefler, Maria E. Bretana, Ramiro Pino, E. Brian Butler, Bin S. Teh

**Affiliations:** a Department of Radiation Oncology, University of Texas Medical Branch at Galveston, Galveston, Texas; b Retina Consultants of Texas, Houston, Texas; c Department of Radiation Oncology, Houston Methodist Hospital, Houston, Texas

## Introduction

Current standard of care for uveal melanoma depends largely on tumor size (Table 1).^1^ Observation is appropriate for many small tumors.^2^ Eye plaque brachytherapy (EPBT) is the preferred treatment for medium size lesions because it is globe-preserving, has comparable survival to enucleation, has a low rate of local failure, and can be successfully salvaged with enucleation.^3^ Enucleation alone is most commonly performed for large lesions because it has been shown that there is no meaningful benefit to neoadjuvant external beam radiation therapy.^4^ Less common approaches for large lesions include EPBT, proton therapy, and stereotactic radiosurgery.

**Table 1:** Table showing tumor size by COMS and NCCN categories

	COMS		NCCN Categories (1)	
	Basal diameter	Apical height	Basal diameter	Apical height
Small	≤ 16 mm	< 2.5 mm	≤ 18 mm	< 2.5 mm
Medium	≤ 16 mm	2.5- 10 mm	≤ 18 mm	2.5- 10 mm
Large	> 16 mm	> 10 mm (or > 8 mm if <2 mm from optic disc)	> 18 mm	> 10 mm (or > 8 mm with optic nerve involvement)

Although current American Brachytherapy Society guidelines stipulate that EPBT may be used for large uveal melanoma if basal diameters do not exceed the limits of brachytherapy and there is ≤5 mm extraocular extension, there are no randomized controlled trials comparing EPBT to enucleation for large uveal melanoma.^5^ A recent large national retrospective study showed that large uveal melanoma treated with EPBT had comparable survival to enucleation.^6^ Genomic data supports this assertion, in that micrometastases have typically already occurred in patients who present with large tumors with aggressive genetic signatures.^7^ Additionally, 2 prior retrospective studies evaluating EPBT for large uveal melanoma showed acceptable local failure rates (6%-9%) at 5 years.^8^^,^^9^

Without custom-building an eye-plaque, only lesions up to approximately 18 mm in basal diameter can be treated with standard 2 mm margins because the largest commercially available I-125 eye-plaque is 22 mm.^1^ Puusaari et al used custom-made plaques up to 25 mm in diameter which covered their largest tumor (25 mm), albeit without standard margins.^8^ Another study by King et al included tumors with basal diameters up to 29 mm and used commercial plaques, counseling patients on potential limits of therapy when tumor size plus margin exceeded plaque size.^9^ In an attempt to offer globe-preservation for uveal melanoma patients with tumors that were at the upper limit of the size of our commercially available plaques, but would have developed unacceptable toxicity, we developed a staged approach for eye-plaque brachytherapy that still provided standard coverage and dosimetry with a more fractionated approach.

## Methods and Materials

### Background

Our institution is a high volume EPBT center with a 0% local failure rate in our case series of 145 patients who had a minimum and mean of 12- and 29-months follow-up.^10^^,^^11^ Our practice is under the direction of a single ocular oncologist (A.C.S.) at a single institution. For treatment planning we perform fusion of images from fundoscopy, B Scan ultrasonography, and thin cut (1 mm) computed tomography (CT). We use Eye Physics EyePlaque Simulator software for 3-dimensional modeling of each patient's eye and tumor, the plaques, precise calculation of suture coordinates for the surgeon, and dosimetry. The calculated algorithm used for dosimetry is a modified version of TG-43 formalism that includes attenuation by the silastic layer and scatter from the gold backing of the plaque.^12^ We prescribe a dose of 85 Gy to the prescription point (either 5 mm or the apical height of the tumor, whichever is greater) for a dwell time of 168 hours with a directive for 95% of the tumor volume to receive 100% of the prescribed dose while keeping maximal scleral dose under 400 Gy. Our intraoperative procedure consists of intraoperative biopsy with real-time verification by an ocular pathologist, placement of an I-125 second- or third-generation Eye Physics gold plaque, and intraoperative ultrasonography for placement verification with or without additional intraoperative plaque localization with transillumination. If necessary, plaques are adjusted after intraoperative ultrasonography to ensure optimal placement. All patients have DecisionDx-UM gene expression assay (Castle Biosciences, Phoenix, AZ), PRAME testing, and NextGeneration sequencing and are followed every 3 to 6 months by our ocular oncologist.

Due to the sophistication of our treatment planning and intraoperative procedures, we have treated many tumors that are classified as large due to apical height. However, our desire to obtain appropriate tumor coverage with margin while maintaining scleral dose constraints has previously prevented us from treating some large-diameter lesions. Thus, we developed a staged technique that enabled us to maintain appropriate target coverage without exceeding scleral dose constraints.

### First patient

**Figure 1:**
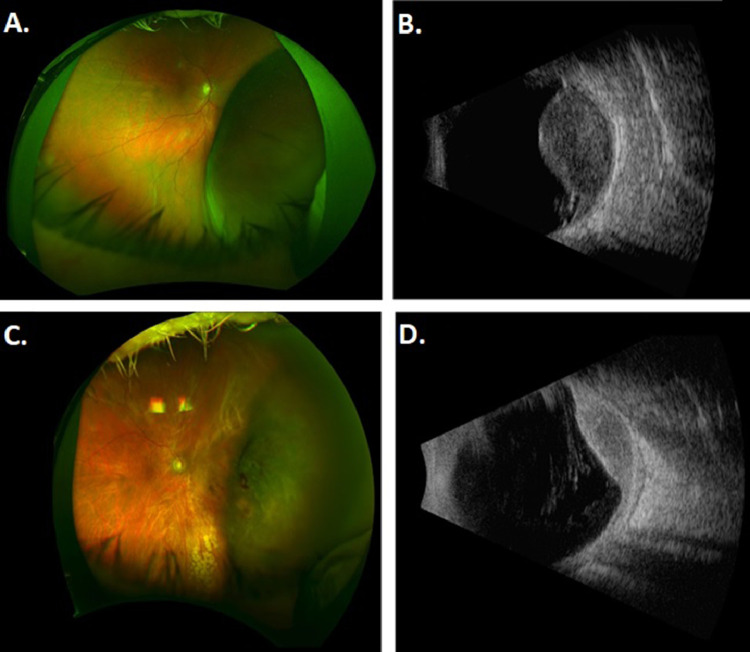
Tumor of patient A. (A) Fundoscopy at presentation. (B) Ultrasonography at presentation. (C) Fundoscopy at last follow-up. (D) Ultrasonography at last follow-up.

**Figure 2:**
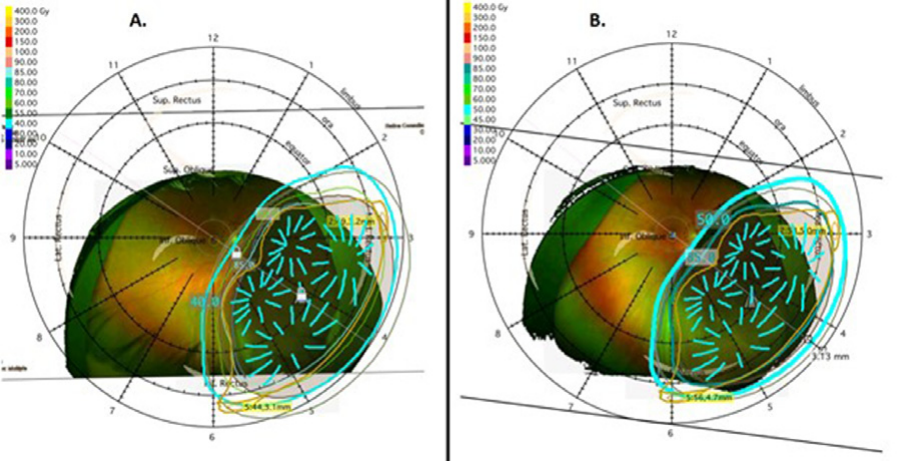
Radiation treatment planning for patient A showing fundoscopy with overlay of eye-plaque (dark yellow), radioactive seeds (cyan lines), and prescribed isodose line (cyan encircling line). (A) Stage 1. (B) Stage 2.

Patient A presented as a 67-year-old man with no past medical history and a 3-week history of decreased vision in his right eye. He had 20/40 vision in his right eye and 20/20 vision in his left eye and fundoscopic examination demonstrated a uveal melanoma from 2:00 to 6:00 in the right eye (Fig. 1A). Ultrasound A/B scan (Fig. 1B) showed a large dome-shaped lesion at 4:00 extending to within 1.1 mm of the optic disc with no extraocular extension and basal diameter of 20.5 mm and a height of 9.9 mm. Our largest commercially available plaque, manufactured by Eye Physics (Eye Physics LLC, Los Alamitos, CA) has a diameter of 23 mm. With this plaque, to reach an apex dose of 85 Gy at a height of 9.9 mm, the maximum scleral dose would have been 543 Gy. In our experience, scleral doses in this range result in severe and immediate toxicity, with postoperative vitreous hemorrhages, severe inflammation, and neovascular glaucoma. The patient was informed that due to the concern for toxicity, his alternative option was enucleation, but he strongly preferred a globe-sparing approach. He received his first plaque brachytherapy insertion in July 2018 using an Eye Physics plaque 2249 fully loaded with 49 I-125 seeds (Fig. 2A). This was implanted for 55 hours and the prescribed dose was 40 Gy to a height of 9.9 mm. The tumor was treated with a 2 mm margin and had 94% of the volume receiving 100% of the prescribed dose and the scleral Dmax was 256 Gy. Biopsy at the time of the procedure showed spindle cell uveal melanoma, class IB PRAME negative. Follow-up 2 months later showed 20/200 vision in his right eye and 20/25 vision in his left eye and fundoscopic examination showed a stable uveal melanoma. Ultrasound A/B scan was not performed before the second implant because inflammation related to the first implant would preclude an accurate height measurement. The second plaque insertion was performed 73 days after the initial insertion, again using Eye Physics plaque 2249 fully loaded with 49 I-125 seeds implanted for 76 hours with a prescription dose of 50 Gy to a height of 10 mm (Fig. 2B). The tumor was treated with a 2 mm margin and had 98% of the volume receiving 100% of the prescribed dose with a scleral Dmax of 328 Gy.

### Second patient

**Figure 3:**
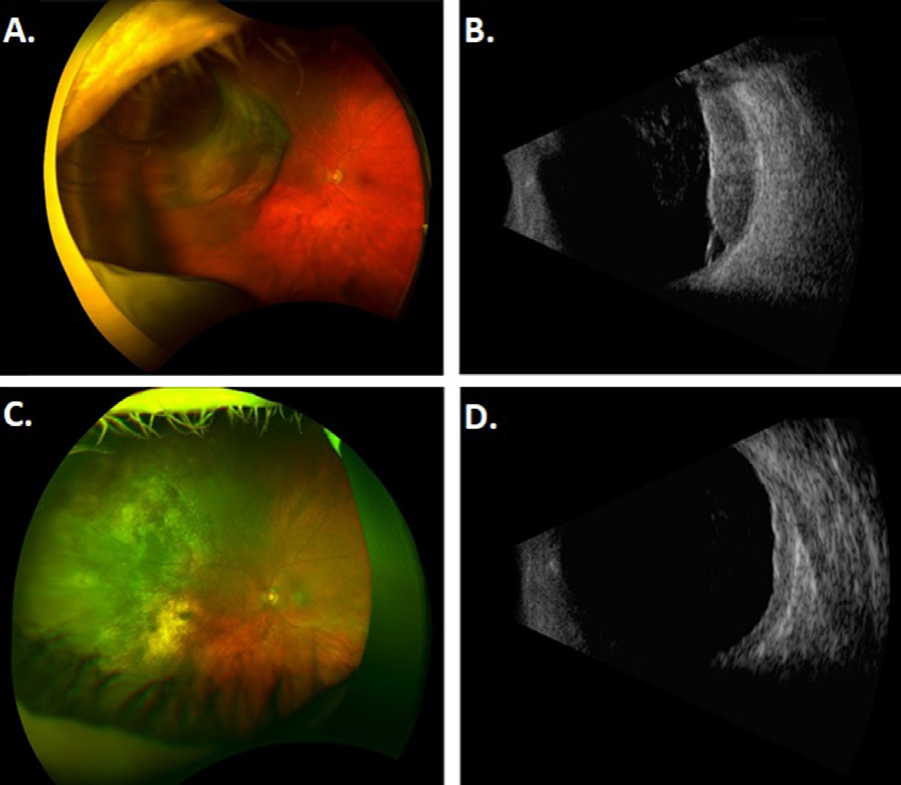
Tumor of patient B. (A) Fundoscopy at presentation. (B) Ultrasonography at presentation. (C) Fundoscopy at last follow-up. (D) Ultrasonography at last follow-up.

**Figure 4:**
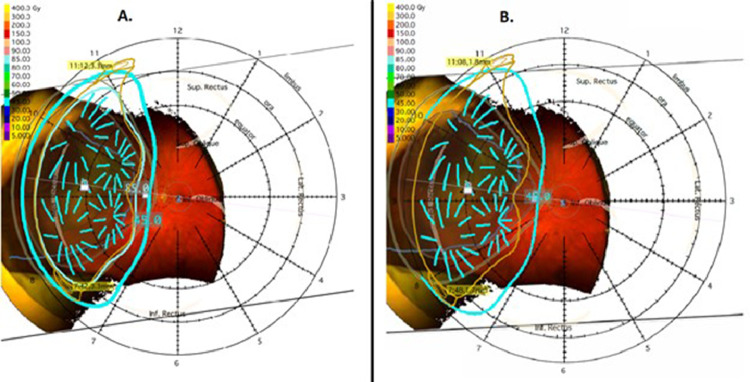
Radiation treatment planning for patient B showing fundoscopy with overlay of eye-plaque (dark yellow), radioactive seeds (cyan lines), and prescribed isodose line (cyan encircling line). (A) Stage 1. (B) Stage 2.

Patient B presented as a 62-year-old man with a past medical history of hypertension and a 2-day history of flashes of light in his left temporal visual field. He had 20/30 vision in his right eye and 20/40 vision in his left eye and fundoscopic examination showed a uveal melanoma from 8:30 to 11:00 in the left eye (Fig. 3A). Ultrasound A/B scan (Fig. 3B) showed a large lobulated shaped lesion from 8:30 to 11:00 in the left eye extending to within 6.2 mm of the optic nerve with no extraocular extension and basal diameter of 21.5 mm and height of 5.4 mm. The patient opted for our staged EPBT approach and received his first plaque brachytherapy procedure in August 2019 using an Eye Physics plaque 2249 fully loaded with 49 I-125 seeds (Fig. 4A). This was implanted for 71 hours and the prescribed dose was 45 Gy to a height of 9.5 mm. Despite a tumor height of 5.4 mm, we had to prescribe to a height of 9.5 mm to achieve adequate tumor coverage because the basal diameter was very large. The tumor was treated with a 2 mm margin with 96% of the volume receiving 100% of the prescribed dose and the scleral Dmax was 268 Gy. Biopsy at the time of the procedure showed spindle cell uveal melanoma, Class 2 PRAME positive. Magnetic resonance imaging (MRI) at the time of diagnosis showed at least 5 hepatic lesions suspicious for metastatic disease, but too small to biopsy. Follow-up 3 months later showed 20/40 vision in his right eye and 20/30 vision in his left eye. The second eye-plaque insertion was performed 106 days after the initial insertion using Eye Physics plaque 2249 fully loaded with 49 I-125 seeds implanted for 71 hours with a prescription dose of 45 Gy to a height of 9.5 mm (Fig. 4B). The tumor had a 2 mm margin with 96% of the volume receiving 100% of the prescribed dose and the scleral Dmax was 267 Gy.

## Results

Patient A tolerated both stages of EPBT without any symptoms or complications. Follow-up 1 month after the second stage demonstrated 20/300 vision in his right eye and 20/20 vision in his left eye. At last follow-up 23 months after the second stage, his vision had improved to 20/50 in the right eye. His fundus examination demonstrated no signs of radiation retinopathy (Fig. 1C) and A/B ultrasonography demonstrated that the lesion had a largest basal diameter of 16.5 mm and a height of 5.2 mm (Fig. 1D, Table 2 ). Recent metastatic screening with chest CT and abdominal MRI was negative for metastatic disease.

**Table 2:** Visual acuity and lesion size for Patient A and Patient B through clinical course.

**Patient A**	OD	OS	Tumor Size on B Ultrasonography
Initial Consultation	20/40	20/20	20.5mm r x 19.5mm c x 9.9mm h
1 wk after Stage 1	20/30	20/20	
2 mo after Stage 1	20/200	20/25	
1 wk after Stage 2	HM	20/25	
1 mo after Stage 2	20/300	20/20	
4 mo after Stage 2	20/100	20/20	20.0mm x r 19.0mm c x 7.7mm h
7 mo after Stage 2	20/80	20/20	18.0mm r x 19.0mm c x 7.5mm h
11 mo after Stage 2	20/200	20/20	17.5mm r x 18.5mm c x 7.0mm h
16 mo after Stage 2	20/50	20/25	16.0mm r x 18.5mm c x 6.7mm h
23 mo after Stage 2	20/50	20/20	16.0mm r x 16.5mm c x 5.2mm h
**Patient B**	OD	OS	Tumor Size on B Ultrasonography
Initial Consultation	20/30	20/40	21.5mm r x 16.5mm c x 5.4mm h
1 wk after Stage 1	20/30	20/400	
3 mo after Stage 1	20/40	20/30	
1 wk after Stage 2	20/40	20/400	
1 mo after Stage 2	20/40	20/40	
2 mo after Stage 2	20/40	20/40	
3 mo after Stage 2	20/40	20/40	13.0mm r x 9.0mm c x 1.6mm h
7 mo after Stage 2	20/40	20/50	

Patient B also tolerated both stages of EPBT without any symptoms or complications. Follow-up 1 month after the second eye-plaque insertion demonstrated 20/40 vision in his right eye and 20/40 vision in his left eye. At last follow-up 11 months after his first procedure, his vision is 20/50 in his left eye and A/B ultrasonography showed the lesion has continued to decrease in size to a largest basal diameter of 13.0 mm and a height of 1.6 mm (Fig. 3C-D, Table 2). His suspicious hepatic lesions have remained stable with the largest lesion measuring 1.3 cm. He continues observation with abdominal MRIs every 3 months.

## Discussion

We have shown that staged EPBT is feasible with acceptable dosimetry and satisfactory early outcomes. Although follow-up for both patients has been relatively short, even if either patient ever needed salvage enucleation at some point, they have both already benefited from months of globe preservation. Globe preservation is desirable because it has better cosmesis with potential visual preservation. Furthermore, patient B's aggressive genomic signature would predict that local therapy will not have an effect on his long-term survival, making globe preservation even more beneficial from a quality of life standpoint. The majority of very large lesions have an aggressive genomic profile, so a globe-conserving approach that maintains vision makes sense for these patients.

Unfortunately, visual preservation after EPBT is poor. In the Collaborative Ocular Melanoma Study (COMS) trial evaluating EPBT for medium size tumors, only 57% of patients had visual acuity better than 20/200 at 3 years.^13^ As expected, visual preservation is even worse in large tumors. In the study by Puusaari et al, only 4% of patients maintained visual acuity better than 20/200 at 3 years.^8^ Factors that have been shown to be associated with poor visual outcomes include age, initial visual acuity, tumor location, tumor height, dose to fovea, and dose to optic disc.^14^, ^15^, ^16^, ^17^, ^18^ Thus, radiation dose is the main modifiable risk factor for visual deterioration. Along these lines, a case series by Saconn et al in which dose was de-escalated to 63 Gy led to 78% of patients with visual acuity better than 20/200 at 5 years and a 10% local failure rate.^19^ Likewise, Perez et al have published data in which 190 patients in the lowest quartile apex dose (<69 Gy) had better visual acuity preservation (70% of patients with visual acuity better than 20/200) without inferior local control or overall survival.^20^

Although dose de-escalation is one method for reducing toxicity, it would be a concerning approach for large tumors. Because large tumors have more cells, fundamental radiobiology dictates that for the same radiation modality a higher dose would be required to achieve the same level of tumor control.^21^ Therefore, instead of dose de-escalation for this subset, we argue that a better method for reducing toxicity while still maintaining local control for large tumors is to split the dose into multiple fractions. From a radiobiological perspective, fractionation reduces late toxicity because it provides time for normal tissue repair.^22^ This notion holds true for all malignancies where the tumor has a higher alpha/beta ratio than the normal tissue.^22^ An in vitro study testing multiple uveal melanoma cell lines detected a median alpha/beta ratio of 10.3 (interquartile range, 8.8-14.1), which approximates that of most other malignancies.^23^ However, ocular melanoma is a slow-growing tumor, which would empirically suggest a much lower alpha/beta ratio. Regardless, assuming an alpha/beta ratio of 3 for normal ocular tissue such as the retina, there is likely a therapeutic benefit to fractionation.

Biologically effective dose (BED) for a low dose rate temporary implant can be calculated using the generalized Lea-Catcheside model.^24^ The equation for this model is BED = D + gD^2^/(α/β), where D represents dose, g is the dose protraction factor that incorporates continuous repair, and α/β is the tissue radiobiologic factor.^24^^,^^25^ For our typical patient who is treated with a single implant, we prescribe 85 Gy over 1 week (168 hours), which equates to a BED of 93.6 Gy. For 2 fractions of 45 Gy over 72 hours that are delivered 90 days apart, the accumulated BED is 101.2 Gy. This calculation excludes repopulation. By comparison, 2 fractions of 40 Gy over 72 hours that are delivered 90 days apart would result in a BED of 88.8 Gy. Therefore, we decided that 2 fractions of 40 Gy would be insufficient but 2 fractions of 45 Gy would be sufficient and presumably enough to account for repopulation.

The technique of splitting a single radiation therapy treatment into 2 fractions to reduce toxicity is not without precedent. A recent randomized phase 2 trial showed that a 2-fraction high-dose-rate brachytherapy approach for low and intermediate-risk prostate cancer results in less urinary toxicity than a single fraction approach.^26^ Similarly, stereotactic radiosurgery for large brain metastasis, which historically was administered in a single fraction is now commonly performed in either 3 fractions delivered every other day or a staged approach with 2 fractions 1 month apart to reduce the risk of brain radionecrosis.^27^^,^^28^

The obvious downside to staged EPBT is the need for 2 plaque insertions. However, the cumulative duration the plaques remain in the eye does not significantly increase because the cumulative prescription dose is similar and dose rate can be maintained. A more concerning theoretical detriment to staged EPBT is the potential for tumor recurrence during the interval between the 2 plaque insertions. This is a malignancy with a relatively long doubling time, however, so we would not expect such an event in an interval of <3 months.^29^ The optimal time interval between the 2 stages remains an open question, but we propose that approximately 2 to 3 months is satisfactory to allow enough normal tissue healing without permitting local tumor recurrence.

The major benefit of this approach is tumor control with reduced ocular toxicity and therefore globe salvage in these patients. The study by King et al found a 3-year radiation retinopathy rate of 38.8% in their population of 158 tumors with a median largest basal diameter of 20.0 mm and a median height of 10.4 mm.^9^ Thus far, our 2 treated patients had satisfactory dosimetry and have fared excellently in terms of toxicity, better than what we would expect from a single stage approach.

There are only 2 patients and follow-up is relatively short. Ideally, we would complete a prospective trial comparing a staged approach to a single-stage EPBT with outcome variables of local treatment failure and toxicity. These 2 cases represent a first step in that direction. In theory, if dose reduction were explored on a larger scale in medium or small tumors and resulted in an unacceptably high local recurrence rate, a staged approach could be considered in that population as well. In our view, this would be a seminal breakthrough in the management of uveal melanoma.

## Conclusions

Staged eye-plaque brachytherapy for large-diameter uveal melanoma is a novel idea we implemented for 2 patients who would have otherwise undergone enucleation. Preliminary results suggest no local recurrence and no appreciable toxicity. Additional exploration is necessary to determine whether any subset of patients may benefit from this globe-sparing approach as standard of care.

## Declaration of Competing Interest

The authors declare that they have no known competing financial interests or personal relationships that could have appeared to influence the work reported in this paper.
